# Effects of Microstimulation in the Anterior Intraparietal Area during Three-Dimensional Shape Categorization

**DOI:** 10.1371/journal.pone.0136543

**Published:** 2015-08-21

**Authors:** Bram-Ernst Verhoef, Rufin Vogels, Peter Janssen

**Affiliations:** 1 Laboratorium voor Neuro- en Psychofysiologie, O&N2, Campus Gasthuisberg, KU Leuven, Leuven, Belgium; 2 Department of Neurobiology, The University of Chicago, Chicago, Illinois, United States of America; University of Verona, ITALY

## Abstract

The anterior intraparietal area (AIP) of rhesus monkeys is part of the dorsal visual stream and contains neurons whose visual response properties are commensurate with a role in three-dimensional (3D) shape perception. Neuronal responses in AIP signal the depth structure of disparity-defined 3D shapes, reflect the choices of monkeys while they categorize 3D shapes, and mirror the behavioral variability across different stimulus conditions during 3D-shape categorization. However, direct evidence for a role of AIP in 3D-shape perception has been lacking. We trained rhesus monkeys to categorize disparity-defined 3D shapes and examined AIP's contribution to 3D-shape categorization by microstimulating in clusters of 3D-shape selective AIP neurons during task performance. We find that microstimulation effects on choices (monkey M1) and reaction times (monkey M1 and M2) depend on the 3D-shape preference of the stimulated site. Moreover, electrical stimulation of the same cells, during either the 3D-shape-categorization task or a saccade task, could affect behavior differently. Interestingly, in one monkey we observed a strong correlation between the strength of choice-related AIP activity (choice probabilities) and the influence of microstimulation on 3D-shape-categorization behavior (choices and reaction time). These findings propose AIP as part of the network responsible for 3D-shape perception. The results also show that the anterior intraparietal cortex contains cells with different tuning properties, i.e. 3D-shape- or saccade-related, that can be dynamically read out depending on the requirements of the task at hand.

## Introduction

Real-life objects are three-dimensional (3D) and we perceive them as such. 3D perception happens despite the flat retinal projections from which it originates. One of the most powerful visual cues for reconstructing the third dimension of objects is binocular disparity. Horizontal binocular disparity refers to the slight difference in position between corresponding points in each eye's image of an object [1]. Previous research suggests that disparity-based depth perception originates from a set of areas in both the dorsal and the ventral visual stream [1–3], in addition to areas in frontal cortex [4–6]. However the precise role of each area in depth perception is still unknown [7].

Several findings indicate an involvement of the ventral visual stream in disparity-defined 3D-shape perception [3,8–14]. For example, neurons in the inferior temporal (IT) cortex encode the shape of disparity-defined 3D shapes [15], their activity correlates with perceptual decisions about 3D shapes during 3D-shape categorization [16], and electrical microstimulation of these IT neurons reliably influences 3D-shape-categorization behavior [17].

On the other hand, areas in the dorsal visual stream have also been implicated in visual categorization and discrimination tasks, thereby opening the possibility of an involvement of dorsal-stream areas in 3D-shape perception [18–23]. One dorsal stream area, the anterior intraparietal area AIP, contains neurons that signal the depth structure of disparity-defined 3D shapes [24,25]. Furthermore, when monkeys are instructed to categorize simple disparity-defined 3D shapes as either convex or concave, the activity of AIP neurons is predictive of the categorization choices and reaction times of these monkeys during task performance [26]. In that same task, the information encoded by AIP neurons about the 3D shape of stimuli depends on the position in depth of the stimulus in a way that mirrors the monkeys' behavioral dependency on position-in-depth during 3D-shape categorization. These response properties of AIP neurons are consistent with a role in 3D-shape perception, yet causal evidence supporting this claim is lacking.

We sought to investigate AIP's role in 3D-shape categorization. We did so by electrically microstimulating clusters of AIP neurons with a similar 3D-shape preference and determining the effect of this stimulation on the behavioral choices and reaction times of monkeys performing a 3D-shape categorization task. We find that microstimulation effects on choices (monkey M1) and reaction times (monkey M1 and M2) depend on the 3D-shape preference of the stimulated site.

## Materials and Methods

### Subjects and surgery

**Fig 1:**
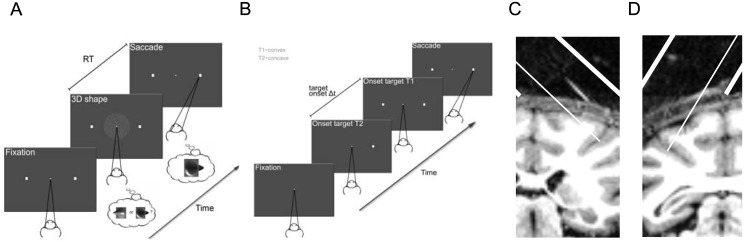
Tasks and recording positions. Tasks and recording procedures are identical to those described in detail previously [26]. (**A**) 3D-shape categorization task. Following a random-duration fixation period, a static random-dot stereogram showing either a convex or concave 3D-shape was presented on a screen. Anytime after stimulus onset, the monkey could indicate whether he/she perceived a convex or concave 3D shape by means of a saccadic eye movement to one of two choice-targets positioned to the left (convex) and right (concave) of the fixation dot. Except when otherwise stated, the same 3D-shape-saccade contingencies were used throughout all stimulation sessions: a leftward saccade for convex shapes and a rightward saccade for concave shapes. Microstimulation was applied on 50% of the trials, randomly chosen. (**B**) Target-onset-asynchrony saccade (TOAS) task. Following a random-duration fixation period, two choice targets (T1 and T2) appeared on the screen with a variable time delay (TOA) between their onsets. Anytime after stimulus onset, the monkey could indicate the choice target that had appeared first by means of a saccade to that target. Choice target T1 and T2 corresponded to convex and concave choices respectively during the 3D-shape categorization task. Microstimulation was applied on 50% of the trials, randomly chosen. (**C**) Coronal magnetic resonance image (MRI) of an AIP recording position in monkey M1. The thick white lines mark the medial and lateral boundary of the recording cylinder. The thin white line illustrates the average electrode position in the anterior lateral bank of the IPS. The short white line lateral of the electrode position is the image of a glass capillary filled with a copper sulphate solution. (**D**) MRI of an AIP recording position in monkey M2.

As described in detail previously [26], one male (M1) and one female (M2) rhesus monkey (*Macaca mulatta*) served as subjects. Both monkeys participated in earlier studies [17,26]. Each monkey received a recording chamber (Crist Instrument) that was positioned over the right (M1) or left (M2) anterior intraparietal sulcus (IPS; Fig 1C and 1D). The recording chambers were tilted with the electrode axis ~40 degrees from vertical, allowing electrodes to penetrate the lateral bank of the IPS approximately orthogonal to the cortical surface. We confirmed that the recordings were made in the anterior part of the lateral bank of the IPS (Horsley-Clark coordinates: M1: -0.6–3.6mm anterior, 13–19mm lateral; M2: 0.8–3.8mm anterior, 12–16mm lateral) by means of structural MRI (0.6 mm slice thickness) using glass capillaries filled with a 1% copper sulphate solution inserted into several grid positions and the pattern of grey-to-white matter transitions. The coordinates of the stimulated AIP regions in our study correspond to regions in which previous studies have shown that cells respond selectively during different grasping actions [27,28]. Furthermore we performed muscimol injections into 3D-shape selective AIP regions in a monkey that did not participate in the present study and observed strong grasping deficits [29].

### Ethics Statement

All surgical procedures and animal care were approved by the KU Leuven Ethical Committee and in accordance with the European Communities Council Directive 2010/63/EU, and all efforts were made to minimize suffering. Animals were anesthetized with ketamine (15 mg/kg, intramuscular) and given atropine (0.05 mg/kg, intramuscular) to reduce salivary fluid production. Recording chambers were implanted under isoflurane anesthesia and aseptic conditions. Prior to surgery, animals were given a preemptive dose of tradonal as analgesic, which was also given for 48 h post-operatively. The animals recovered for four weeks before resuming training. The monkeys were not noticeably disturbed by the recording chamber post-recovery, and the recording chamber did not affect the monkeys' ability to move, see, or perform self-care activities. No animal was sacrificed for the purpose of this experiment.

Both animals were pair housed in standard 12:12 light-dark cycle, were given food (primate biscuits) *ad libitum* supplemented with nuts, raisins, prunes and fruits if necessary, received their daily water supply during the experiments, and their weight was measured daily. Animals obtained new toys on a regular basis and were admitted to an enriched (robes, tree trunks, etc.) "play cage" whenever possible.

### Stimuli and Tasks

#### 3D-shape categorization task

Stimuli and task procedures correspond to those described in detail previously [26]. We trained monkeys to discriminate convex and concave 3D shapes. The stimulus set consisted of static random-dot stereograms with 8 different 2D-shape outlines (e.g. circle, ellipse, square, etc.; see Fig S1 in Verhoef et al. 2012; size: ~ 5°). Stimuli were presented foveally on a grey background. Previous studies have shown that foveally-presented stimuli are generally effective in activating AIP neurons [24,30]. The depth structure of the stimulus was defined solely by horizontal disparity as a two-dimensional radial basis Gaussian surface (standard deviation = 48 pixels, 0.96°) that could be either convex or concave (maximal disparity amplitude: 0.15°). The dots consisted of Gaussian luminance profiles (width: 7 pixels; height: 1 pixel; horizontal standard deviation: 0.7 pixels; 1 pixel ≈ 0.02°). For each dot, the mean of the Gaussian luminance profile could be positioned along a continuous axis resulting in perceptually smooth stereograms with sub-pixel resolution. Stimuli were presented at 3 positions in depth, i.e. before (Near), behind (Far) or at (Fix) the fixation plane (±0.23° depth variation). Stimuli were presented dichoptically using a double pair of ferroelectric liquid crystal shutters (two superimposed shutters in front of each eye; Displaytech). The four shutters operated at 60Hz and were synchronized with the vertical retrace of the display monitor (vertical refresh rate, 120Hz) equipped with a fast-decay P46 phosphor (VRG). Each eye was therefore stimulated at 60Hz. There was no measurable crosstalk between the eyes. The viewing distance was 86 cm. Task difficulty, defined as stereo-coherence, was manipulated by varying the percentage of dots defining the surface. Dots that were not designated as defining the surface were assigned a disparity drawn randomly from a uniform distribution (support = [-0.50°, 0.50°]). For each experiment we used 20 different random-dot patterns per signal strength. Each trial started with a prestimulus fixation interval, the duration of which was randomly selected from an exponential distribution (mean = 570ms, minimum duration = 250ms, maximum duration = 1500ms). Following the fixation period, a stimulus with a randomly chosen shape (convex or concave), position-in-depth (Far, Fix, Near) and stereo-coherence (0%, 15%, 20%, 30%, 100%) was presented on the monitor. After stimulus onset, the monkey was free to indicate its choice at any time by means of a saccade to one of two choice targets located at 6° eccentricity from the fixation point (Fig 1A). Monkeys maintained fixation (fixation window < 1.5° on a side) on a small fixation point until they initiated their choice saccade. The stimulus was extinguished once the monkey left the fixation window. Choice-targets were visible throughout the trial until one of the choice targets had been fixated for 300ms. Correct responses were followed by a liquid reward (apple juice). Except when otherwise stated, the same 3D-shape-saccade contingencies were used throughout all stimulation sessions: a leftward saccade for convex shapes and a rightward saccade for concave shapes. The 0% signal strength trials were randomly rewarded with a 0.5 probability.

#### Target-onset-asynchrony saccade task

As described in detail previously [26], each trial of the target-onset-asynchrony saccade (TOAS) task started with a prestimulus interval, the duration of which was randomly selected from an exponential distribution (mean = 570ms, minimum duration = 250ms, maximum duration = 1500ms). Following the prestimulus period, two choice targets appeared on the screen with a variable time delay (TOA) between their onsets. The monkey was rewarded for making a saccade to the choice target that appeared first (Fig 1B). Each trial, a TOA was randomly chosen, ranging between 8ms and 150ms. This procedure assured that on any trial the monkey could neither predict the location of the first target nor the TOA. The location of the choice targets matched those of the categorization task, i.e. 6° eccentric from the fixation point.

### Recording of neuronal and eye position signals and microstimulation parameters

As described in detail previously [16,26], extracellular recordings were made using tungsten microelectrodes. Microstimulation caused the impedance of the electrodes to drop by ~0.4 MΩ over the course of a stimulation session. Therefore we started each recording sessions with a relatively high impedance electrode (~0.7–1.2 MΩ at 1 kHz; FHC), so that we could still record high-quality multi-unit activity (MUA) (~0.3–0.8 MΩ at 1 kHz) after finishing the microstimulation experiments (i.e. 3D-shape categorization and the TOAS task). The MUA measured in the post-stimulation recordings was used to ascertain that the 3D-shape preference of the neurons at the stimulated site had not changed during microstimulation, and to measure choice probabilities. MUA was recorded by setting a threshold approximately three standard deviations below the average noise level. Electrical pulses for microstimulation purposes were delivered using a pulse generator (DS8000; World Precision Instruments) in series with an optical stimulus isolator unit (DLS100; World Precision Instruments). Stimulation consisted of bipolar current pulse trains of 35 μA (other currents were used as well; see Results) delivered at 200Hz. We used biphasic (cathodal pulse leading) square-wave pulses with a pulse duration of 0.2 ms and 0.1 ms between the cathodal and anodal pulses (total pulse duration = 0.5 ms). During the 3D-shape categorization and the TOAS task, stimulation was applied on half of the trials, chosen randomly. Thus trials with and without stimulation were randomly interleaved. During the 3D-shape categorization task, stimulation started 50ms after stimulus onset and ceased when the monkey's gaze left the fixation window to indicate its choice. During the TOAS task, stimulation started at stimulus onset and ceased when the monkey's gaze left the fixation window to indicate its choice. These timing parameters and the pattern of reaction times for each task (reaction times were about 50 ms shorter during the TOAS task, compared to the categorization task) resulted in approximately equal amounts of stimulation during both tasks for either monkey. The mean duration of the microstimulation for monkey M1 was 193 ms during the TOAS-task and 209 ms during the 3D-shape categorization task. The mean microstimulation duration for monkey M2 was 275 ms during the TOAS-task and 272 ms during the categorization task. The positions of both eyes were sampled at 500 Hz using an EyeLinkII (SR Research) system.

### Recording procedure

We employed recording procedures identical to those of a previous microstimulation study in IT [17] and a recent study in AIP [26]. We started each session by sampling AIP along electrode penetrations approximately orthogonal to the cortex in steps of ~100–150μm. For each electrode position in a penetration, we then first selected the optimal (within our stimulus set) 2D-shape outline (e.g. circle, ellipse, square, etc.) for the multiunit activity of that site, using a passive fixation task. With the optimal 2D-shape outline thus chosen, we tested the 3D-shape selectivity of a site by presenting 100% stereo-coherent concave and convex 3D shapes at one of three different positions in depth: in front of the fixation plane (Near), at the fixation plane (Fix), or behind the fixation plane (Far). The electrode was subsequently retracted to the center of the 3D-shape selective cluster where we again verified that the MUA still displayed the same 3D-shape preference before starting the 3D-shape categorization task. We used the optimal 2D-shape outline for the MUA at the cluster center throughout the 3D-shape categorization task. We adopted the following criterion for defining a 3D-shape selective cluster: The center-position of a cluster had to be neighbored by MUA positions having the same 3D-shape selectivity for at least 125μm in both directions (i.e. upward and downward). Similar criteria have been used previously [17,18,22,31]. After each microstimulation session, we ensured that the 3D-shape preference of the MUA had not changed. In order to maximize the number of trials in the main experiment (i.e. 3D-shape categorization and TOAS with and without microstimulation) and to minimize the cortical damage inflicted upon the positions with significant 3D-shape selectivity, we did not sample the entire extent of a 3D-shape selective cluster. The existence of clustering of AIP neurons with a similar 3D-shape preference has been described in a previous study [26].

### Data Analysis

Following procedures described in detail previously [16,26], a site was considered 3D-shape selective if a significant main effect of 3D shape (convex versus concave) was observed in an ANOVA with 3D shape and position-in-depth as factors (p<0.05). The preferred shape of a 3D-shape selective site was defined as the shape with the highest average MUA in the stimulus interval ([100ms, 800ms]; 0 = stimulus onset) as measured during a fixation task (see above).

We used *d’* as a measure of the 3D-shape selectivity of a site [16,26]. The signed *d'* is defined as $d'=\frac{{\bar{X}}_{convex}-{\bar{X}}_{concave}}{\sqrt{\frac{S_{convex}^{2}+S_{concave}^{2}}{2}}}$, where ${\bar{X}}_{convex}$ and ${\bar{X}}_{concave}$ are the mean multiunit responses to convex and concave 3D stimuli respectively, and $S_{convex}^{2}$ and $S_{concave}^{2}$ are the variances of the neuronal responses to convex and concave 3D stimuli respectively. Positive and negative values indicate convex and concave tuning respectively.

Reaction times (RTs) were estimated as follows [16,26]: The horizontal eye-traces of each trial were first low-pass filtered (cutoff = 40Hz) to remove high-frequency noise [32]. The resulting time series ${\vec{x}}_{t}$ was transformed into velocities using the transformation ${\vec{v}}_{n}=\frac{{\vec{x}}_{n+2}+{\vec{x}}_{n+1}-{\vec{x}}_{n-1}-{\vec{x}}_{n-2}}{6\Delta t}$ (Δ*t* = sampling period), which represents a moving average of velocities and serves to suppress noise. The RT was defined as the time point relative to stimulus onset of the first of five consecutive velocities for which the speeds exceeded 50 degrees/s in the same direction. RTs were square root transformed before being entered into an ANOVA.

We used logistic regression to model the behavioral data as a function of stereo-coherence or TOA and the application of microstimulation during a trial [17,18] using the following function (glmfit; Matlab R2009a):$P=\frac{1}{1+\text{exp}[-\beta_{0}+(\beta_{1}\cdot I)+(\beta_{2}\cdot x))]}$, where *P* denotes the probability of a preferred choice, *x* represents the level of stereo-coherence or TOA and *I* is a binary variable which takes the values 1 and 0 on stimulated and non- stimulated trials respectively. The *β*_0_- and *β*_2_-coefficients measure the response bias and slope respectively. The *β*_1_-coefficient measures the effect of microstimulation on the monkey’s response bias. The shift of the psychometric function due to microstimulation was formalized as *β*_1_ / *β*_2_. This model, in which the effect of microstimulation was modeled solely as a horizontal shift or bias, was used throughout all analyses presented here. However, we obtained very similar results by fitting a logistic model that allowed for microstimulation-induced slope changes. In this extended model, we added (*β*_3_ *x I*) to the linear exponent and fitted the model as before. The extended model was also used for plotting purposes.

## Results

We sought to examine the role of the anterior intraparietal area (AIP) in 3D-shape categorization. As described in detail previously [16,26], we trained two rhesus monkeys (M1 and M2) to categorize 3D shapes as either convex or concave. Static random—dot stereograms portrayed either a convex or concave surface which was presented at one of three positions in depth, i.e. in front of (Near), behind (Far) or within (Fix) the fixation plane. This procedure requires the subject to rely on depth variations within the stimulus (i.e. disparity gradients or curvature) and discourages perceptual strategies that are based on position-in-depth information (i.e. “near” or “far” decisions; see [16,26]). Task difficulty was manipulated by varying the percentage of dots defining the 3D surface, henceforth denoted as the percent stereo-coherence (Materials and Methods). The monkeys could indicate their choice at any time after stimulus onset by means of a saccadic eye movement to one of two choice-targets (Fig 1A). This procedure delimits the perceptual-decision process more precisely in time, and allowed us to measure reaction times (RTs; see Materials and Methods). We have previously shown that several aspects of the neuronal activity in AIP show correlations with the behavior of monkeys while they categorize 3D shapes as convex or concave [26]. Here we examine the causal role of AIP in 3D-shape categorization using electrical microstimulation as a tool.

### Microstimulation in clusters of 3D-shape selective AIP neurons

We electrically microstimulated in the centers of 60 clusters of 3D-shape selective AIP neurons displaying similar 3D-shape preferences (M1: N = 16; M2: N = 44 different penetrations). The locations and distribution of the stimulated 3D-shape selective sites are shown in Fig 1C and 1D in [26]. We start by describing the results of microstimulation in AIP of monkey M1.

**Fig 2:**
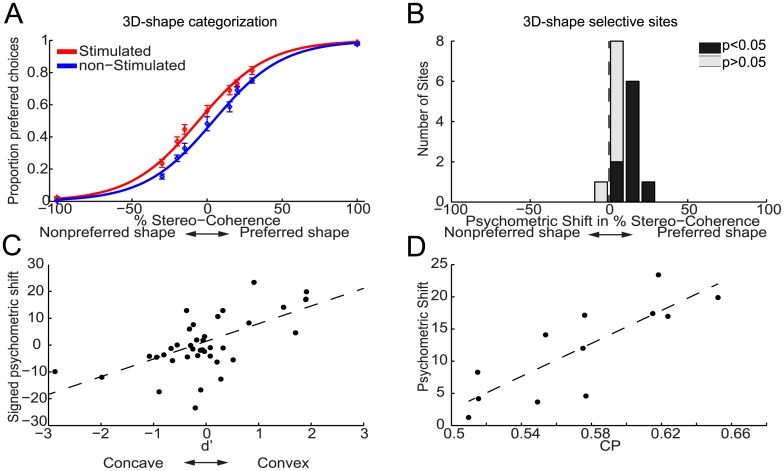
Effect of electrical stimulation in AIP on the choices of monkey M1 during 3D-shape categorization. (**A**) shows the average proportion of choices (±SEM) that matched the preferred shape of the 3D-shape selective site (preferred choices), displayed as a function of stereo-coherence for trials with (red) and without (blue) microstimulation. Positive and negative stereo-coherences relate to the preferred and non-preferred shape, respectively. Solid lines show the fitted psychometric functions. Microstimulation shifted the psychometric function towards a greater number of preferred choices by an average of 10.2%. (**B**) Histogram of microstimulation effects (N = 16 3D-shape selective sites) expressed as a shift of the psychometric function in terms of percent stereo-coherence. Positive values are used for psychometric shifts towards an increase in preferred choices. Black bars indicate sites with a significant shift of the psychometric function due to microstimulation (p<0.05). (**C**) Relationship between selectivity and microstimulation effect at a given site (r = 0.63, p<0.001; N = 16 selective sites; N = 23 nonselective sites). Positive and negative psychometric shifts denote shifts towards more convex and concave choices respectively. Signed d’-values are used to denote the 3D-shape preference of the MUA-sites, with positive and negative values indicating convex and concave preferences respectively. (**D**) Relationship between the CP and microstimulation effect at a given site (r = 0.82, p = 0.001; N = 12). The black dashed lines are robust regression lines.

Fig 2A plots the average proportion of choices favoring the preferred 3D shape of the 3D-shape selective sites, i.e. preferred choices, as a function of stereo-coherence for trials with (red) and without (blue) microstimulation for monkey M1. Percentage stereo-coherence is defined as the percentage of random dots making up the 3D shape (Materials and Methods). By convention, positive stereo-coherences refer to the preferred 3D shape of the neuronal activity of the stimulated site, while negative values relate to the nonpreferred shape of the responses of the 3D-shape selective site. This plot shows that microstimulation in AIP on average increased the proportion of preferred choices. We used logistic regression analysis (average goodness of fit, R^2^, across sites = 0.94; see Materials and Methods) to quantify the effect of microstimulation on the monkeys' choice behavior. The fitted logistic functions are shown in Fig 2A and reveal a clear leftward shift, i.e. towards a higher number of preferred choices, of the psychometric function on trials *with* (red solid line) compared to those *without* (blue solid line) microstimulation.

We quantified the microstimulation-induced horizontal shift of the psychometric function as the proportion of coherent dots (% stereo-coherence) that must be added to the random-dot stereograms to produce a comparable shift in behavior (see Materials and Methods). Fig 2B shows a histogram of the psychometric shifts, expressed as percent stereo-coherence, observed over all 3D-shape selective sites in monkey M1. We observed a significant positive shift (p<0.05; Wald test) towards more preferred choices in 9 out of 16 (~56%) 3D-shape selective sites (black bars in Fig 2B). The average shift of 10.2% stereo-coherence in the direction of more preferred choices was significantly different from zero (p<0.0001, t-test). Microstimulation induced significant psychometric shifts towards an increased number of preferred choices in 6 out of 7 convex-selective sites (average shift = 15%) and in 3 out of 9 concave-selective sites (average shift = 6.5%). The association between the 3D-shape preference of a site and the direction of the psychometric shift due to microstimulation was significant when the analysis was restricted to all sites for which we observed a significant stimulation-induced shift of the psychometric function (N = 9, p = 0.006; Fisher-exact test), or when all 3D-shape selective sites were included (N = 16; p = 0.003). Microstimulation significantly biased the monkey’s choices towards more preferred choices at each of the three stimulus positions-in-depth (p<0.001, t-test). Hence electrical stimulation of convex-preferring neurons in AIP increased the number of convex choices and stimulation of concave-preferring neurons increased the number of concave choices.

We also stimulated in 23 sites not selective for 3D-shape, recorded at the same grid positions as the 3D-shape selective sites. These nonselective sites were neighbored by MUA-positions with no 3D-shape selectivity for at least 125μm in either vertical direction (up- or downwards). For the nonselective sites, we observed significant choice biases due to microstimulation in 6 out of 23 sites (3 towards more convex and 3 towards more concave choices) but the average shift of 1.3% towards more concave choices did not differ significantly from zero (p = 0.48, t-test). Thus microstimulation effects in AIP sites not selective for 3D shape were scarce and did not systematically favor either choice. These findings further demonstrate that the choice-related microstimulation effects in the 3D-shape selective sites of monkey M1 are not caused by factors unrelated to the 3D-shape preference of the stimulated neurons, because such nonspecific effects would be apparent in nonselective sites as well.

Fig 2C plots the shift of the psychometric function against the 3D-shape selectivity of the MUA measured at each stimulation site using a fixation task (see Materials and Methods). For this purpose, negative and positive psychometric shifts denote shifts towards more concave and convex choices respectively. Signed d’-values measure the 3D-shape selectivity of the MUA-sites, with negative and positive values indicating concave and convex preferences respectively (see Materials and Methods). We observed a significant correlation between the signed d’ and the signed psychometric shift (r = 0.63, p<0.001, Fisher-Z test).

We additionally found a correlation between the cluster size, measured orthogonal to the cortical surface, and the magnitude of the microstimulation effect (r = 0.59; p = 0.016, Fisher-Z test), indicating that microstimulation of larger clusters increased the proportion of preferred choices to a greater extent.

In 12 experimental sessions we microstimulated and measured choice probabilities (CP) at the same site. Choice probabilities measure the trial-by-trial correlation between the neuronal activity and the behavioral choices of the monkey during task performance [33]. We have previously shown that AIP contains decision-related neuronal activity during 3D-shape categorization [26]. As can be seen in Fig 2D, we observed a strong correlation of 0.82 between the choice probability of a site and the (unsigned) microstimulation-induced shift of the psychometric function (p = 0.001, Fisher-Z test). This correlation was still significant after controlling for each variable's correlation with the selectivity (d'; partial correlation = 0.79; p = 0.004) of a site. Thus sites with higher correlations between spiking activity and behavioral choice are also characterized by larger choice biases due to microstimulation.

**Fig 3:**
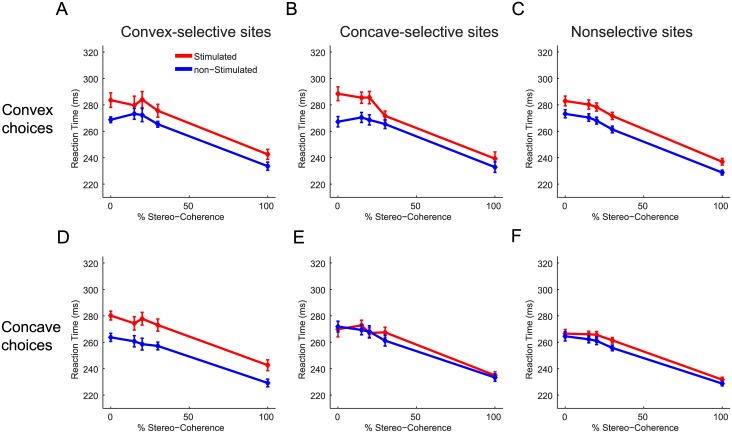
Effect of electrical stimulation in AIP upon the reaction times of monkey M1 during 3D-shape categorization. Average reaction times as a function of stereo-coherence for stimulated (red) and non-stimulated (blue) trials. Microstimulation delayed the execution of choices, especially convex choices. (**A**) Average RTs for convex choices when stimulating in convex-selective sites. (**B**) Average reaction times for convex choices when stimulating in concave-selective sites. (**C**) Average reaction times for convex choices when stimulating in nonselective sites. (**D**) Average reaction times for concave choices when stimulating in convex-selective sites. (**E**) Average reaction times for concave choices when stimulating in concave-selective sites. (**F**) Average reaction times for concave choices when stimulating in nonselective sites. Error bars indicate ±SEM.

Previously we have shown that microstimulation in 3D-shape selective clusters in the inferotemporal (IT) cortex of monkey M1 decreased the average RT for preferred choices while increasing the average RT for nonpreferred choices [17]. This RT-pattern is to be expected if microstimulation increases the evidence in favor of the preferred shape of the stimulated neurons. Fig 3 shows the average RT as a function of stereo-coherence for stimulated (red) and nonstimulated (blue) trials for each combination of choice (convex or concave) and 3D-shape preference (convex-, concave- or nonselective). Consistent with findings in IT, we observed that microstimulation in AIP delays nonpreferred choices (Fig 3B and 3D; mean delay = 14ms; p<0.01, ANOVA). In contrast to IT, however, microstimulation in AIP tended to delay preferred choices: Convex choices for convex-selective sites were significantly delayed by an average of 10.5ms (Fig 3A; p = 0.004, ANOVA) and concave choices for concave-selective sites were slightly, but not significantly, delayed by ~2ms (Fig 3E; p>0.05, ANOVA). Thus on convex-selective sites microstimulation delays either choice (p>0.05 for the interaction between stimulation and choice, ANOVA) and for concave-selective sites, delays were observed for convex choices but little or not at all for concave choices (p = 0.007 for the interaction between stimulation and choice, ANOVA). For sites not selective for 3D shape, significant delays were observed for either choice (p<0.02, ANOVA) but the delay was strongest for convex choices (Fig 3C and 3F; interaction: p = 0.01, ANOVA). Therefore, despite consistent effects on the choices of monkey M1, microstimulation did not accelerate preferred choices but tended to delay choices, particularly convex choices.

**Fig 4:**
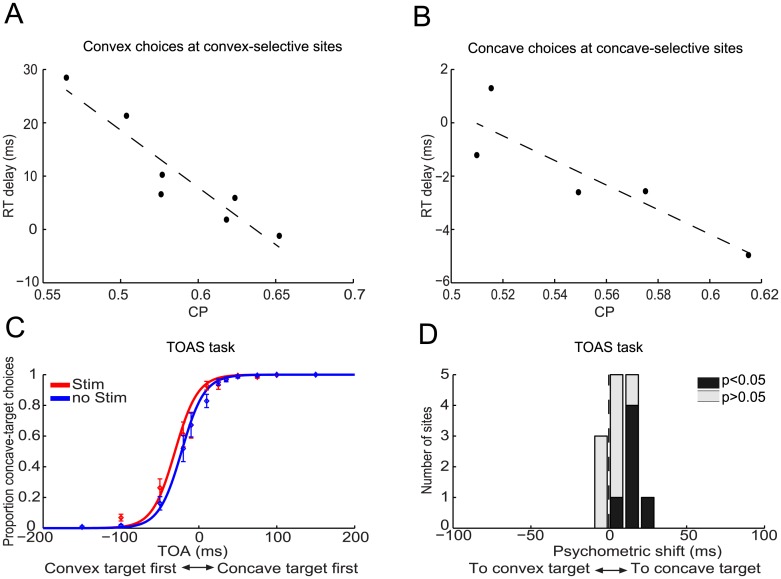
Examining the stimulation-induced RT delays for preferred choices of monkey M1. (**A, B**) Scatter plot of the choice probability versus the delay induced by microstimulation for preferred choices. (**A**) Convex choices with the electrode positioned in convex-selective sites (r = -0.96, p<0.001; N = 7). (**B**) Concave choices with the electrode positioned in concave-selective sites (r = -0.91, p = 0.034; N = 5). The dashed lines are robust-linear regression lines. (**C**) shows the average proportion of choices (±SEM) during the TOAS task that were in the direction of the choice target used for concave choices during the 3D-shape-categorization task for trials with (red) and without (blue) microstimulation. Positive target-onset asynchronies (TOAs) indicate trials on which the (right) concave choice target appeared first. Negative TOAs indicate trials on which the (left) convex choice target appeared first. Solid lines show the fitted psychometric functions. Microstimulation during the TOAS task increased saccades to the right, i.e. towards the concave choice target. (**D**) Histogram of microstimulation effects (N = 14) during the TOAS task. Stimulation effects are expressed as the additional delay (TOA in ms) that would need to be added to the delay between the two targets on trials without microstimulation in order to obtain the behavioral performance actually observed on trials with microstimulation. For example, a delay of 9ms means that microstimulation during the TOAS task had the same effect on the monkey's performance as one would obtain by presenting the (right) concave choice target 9ms earlier than the (left) convex target. Black bars indicate sites showing significant shifts of their psychometric functions due to microstimulation (p<0.05).

Interestingly, microstimulation of sites with larger choice probabilities led to considerably smaller delays for preferred choices. Fig 4A shows that stimulation-induced delays as large as ~30ms disappeared for convex-selective sites with the largest choice probabilities. The correlation between the choice probability and the delay due to microstimulation was highly negative (r = -0.96, p<0.001, Fisher-Z test; N = 7), even after controlling for both variables' correlation with the microstimulation-induced choice effects (partial correlation = -0.89, p = 0.019). Microstimulation in concave-selective sites with the largest choice probabilities even slightly accelerated concave choices (Fig 4B). The correlation between choice probability and the delay due to microstimulation was significantly negative (r = -0.91, p = 0.034; partial correlation = -0.87, p = 0.12; N = 5). There was no significant correlation between a site’s choice probability and the delays for nonpreferred choices (p>0.05). The negative correlation between choice probabilities and reaction-time delays suggest that the reaction-time delays for preferred choices were caused by a source of activity unrelated to the 3D-shape preference of a site. Moreover, these results can be explained by assuming that when microstimulation is applied to sites with a relatively high impact on 3D-shape categorization, as characterized by larger choice probabilities, stimulation-induced 3D-shape related activity better withstands the adverse effects of that other source of interfering activity. One potential source of such interference is activity related to saccade planning which, as shown in a previous study [26], is present in AIP and could therefore also be activated during microstimulation.

The fact that concave choices were generally delayed less than convex choices suggests that the induced saccade-related activity had a direction-preference that corresponded to the direction of the concave choice target (ipsilateral hemifield). To verify this, we stimulated AIP while the monkey executed a target-onset-asynchrony-saccade task wherein the subject was instructed to detect which of two targets—presented with a variable time delay between the two—appeared first, by making a saccade to that target (Fig 1B) (TOAS task; Materials and Methods). Stimulations during the TOAS task were made in grid positions previously associated with 3D-shape selective AIP sites. As predicted by the RT-results of the 3D-shape categorizaton tasks, we observed that microstimulation significantly biased monkey M1’s choices towards making eye movements in the direction of the choice target that was used for concave choices during the 3D-shape categorization task (Fig 4C). We quantified the stimulation-induced psychometric shift during the TOAS-task as the additional delay that should be added to the delay between the two choice targets on trials without microstimulation in order to obtain behavioral performance similar to that during trials with microstimulation (Materials and Methods). Positive shifts are used for stimulation-induced biases in the direction of the concave choice targets and negative shifts for stimulation-induced biases towards the convex choice target. The average positive shift of 8.7ms revealed a significant bias towards the position of the concave choice target (p = 0.005, t-test; N = 14) and the six sites with a significant microstimulation effect all displayed biases towards the concave choice target (Fig 4D).

Next we considered all sites for which microstimulation increased the number of convex choices during the 3D-shape categorization task (N = 7: 3 convex selective, 4 nonselective) and correlated the average RT-delay for convex and concave choices with each site’s stimulation-induced shift during the TOAS-task. The results show a correlation of 0.86 (p = 0.01, Fisher-Z test) for convex choices and 0.76 (p = 0.048) for concave choices. Hence, larger biases in the direction of the concave-choice target during the TOAS-task are associated with longer response delays during the 3D-shape categorization sessions in which stimulation increased the number of convex choices. This suggests that convex responses will be delayed during 3D-shape categorization when microstimulation causes greater interference favoring the concave choice target, because the induced "saccadic" activity (toward concave) opposes the induced 3D-shape-selective activity (toward convex). Note that for three convex-selective sites in which microstimulation significantly (p<0.05, Wald test) increased the number of *convex* choices during 3D-shape categorization (mean shift = 14%), a significant *concave*-choice bias was observed in two sites during the TOAS-task (mean shift = 15.5ms, p<0.002 for each site) and no choice bias in the third site. The different choice biases in the two tasks show that an association between the 3D-shape preference and the saccade-direction preference of the stimulated site did not cause the consistent microstimulation effects on the choices of M1 during 3D-shape categorization. In the subset of the data for which microstimulation increased the number of concave choices during the 3D-shape-categorization task (N = 7: 1 concave selective, 6 nonselective), the correlation between the TOAS-shift and the response delay during 3D-shape categorization did not differ significantly from zero (convex choices: r = 0.47; concave choices: r = 0.24; p>0.05, Fisher-Z test). Note that microstimulation can also boost saccadic activity favoring other directions, as is shown below for monkey M2, which may explain the residual response delays for, e.g., the concave choices on concave-selective sites.

Taken together these findings demonstrate that microstimulation in 3D-shape selective clusters in AIP of monkey M1 activated more than one process. The dominant process increased the evidence in favor of the preferred shape of the stimulated neurons while the other process, potentially related to saccade planning, interfered with this dominant process, thus producing response delays.

**Fig 5:**
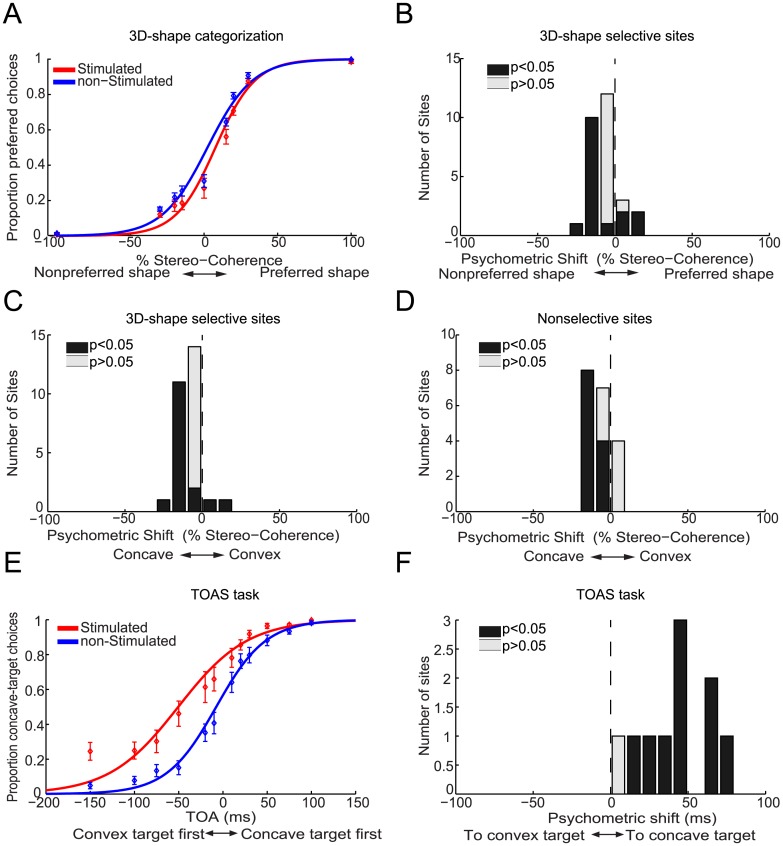
Effect of electrical stimulation in AIP on the choices of monkey M2. 3D-shape categorization task: (**A**) shows the average proportion of choices (±SEM) that matched the preferred shape of the 3D-shape selective site (preferred choices) as a function of stereo-coherence for trials with (red) and without (blue) microstimulation. Positive and negative stereo-coherences relate to preferred and non-preferred shapes, respectively. Solid lines show the fitted psychometric functions. Microstimulation shifted the psychometric function towards a higher number of nonpreferred choices. (**B**) Histogram of microstimulation effects (N = 28 3D-shape selective sites) expressed as a shift of the psychometric function in terms of percent stereo-coherence. Positive values express psychometric shifts towards an increase in the number of preferred choices. (**C**) Histogram of microstimulation effects expressed as a shift of the psychometric function in terms of percent stereo-coherence. Positive and negative values are used for psychometric shifts towards more convex or more concave choices respectively. Microstimulation increased the number of concave choices. (**D**) Same as (**C**) but for nonselective sites (N = 19). TOAS task: (**E**) shows the average proportion of choices (±SEM) during the TOAS task in the direction of the choice target used for concave choices during the 3D-shape-categorization task for trials with (red) and without (blue) microstimulation. Positive TOAs indicate trials where the (right) concave choice target appeared first. Negative TOAs indicate trials where the (left) convex choice target appeared first. Solid lines show the fitted psychometric functions. Microstimulation during the TOAS task strongly increased the number of saccades towards the concave choice target. (**F**) Histogram of microstimulation effects (N = 10) during the TOAS task. Stimulation effects are expressed as the additional delay (TOA in ms) that would need to be added to the delay between the two targets on trials without microstimulation in order to match the behavioral performance that was actually observed on trials with microstimulation. Microstimulation had an effect equivalent to presenting the concave choice target 43ms before the convex choice target. Black bars indicate sites displaying significant shifts of their psychometric functions due to microstimulation (p<0.05).

In contrast to monkey M1, microstimulation in 3D-shape selective clusters in AIP of monkey M2 did not increase the number of preferred choices (Fig 5A). The average stimulation-induced psychometric shift was negative (-5.6%, p<0.001, t-test, N = 28), indicating that microstimulation increased the number of nonpreferred choices (Fig 5B). This can be explained as follows: we encountered considerably more convex-selective clusters in AIP of monkey M2 (N = 25 convex clusters, N = 3 concave clusters). This convexity-bias was also observed in the IT cortex of this monkey [17]. In both convex- and concave-selective clusters, microstimulation increased the number of concave choices (mean shift = 7% for convex- and concave-selective clusters). Accordingly, an increase of the number of concave choices in predominantly convex-selective sites gives rise to an average increase in nonpreferred choices. This is further illustrated in Fig 5C where microstimulation effects are sorted according to whether stimulation increased the number of convex (positive shifts) or concave (negative shifts) choices. The average shift towards more concave choices was -7.2% (p<0.001, t-test). We observed an identical stimulation-induced concave response bias in 19 AIP sites not selective for 3D shape (Fig 5D; mean = -7.2%, p<0.001). In view of the saccade-related stimulation effects in monkey M1 mentioned above, we reasoned that microstimulation in AIP of monkey M2 might have activated stronger interfering processes that drove the monkey’s choice primarily in the direction of the concave choice target (contralateral visual field for this monkey). Therefore we examined the effects of microstimulation while monkey M2 performed the TOAS-task. Fig 5E shows that microstimulation strongly biased monkey M2’s saccades in the direction of the concave choice target. The stimulation effect was significant in 9 out of 10 sessions (p<0.05, Wald test) and the shift of the psychometric function for the TOAS-task averaged 43ms (p<0.001, bootstrap test). Monkey M2’s bias towards the concave choice target during the TOAS-task was significantly stronger than that in monkey M1 (M1: 8.7ms; M2: 43ms; p<0.001, permutation test). The concave-direction bias during the TOAS-task tended to correlate with the concave-bias during 3D-shape categorization (r = 0.52, p = 0.12, N = 10; see also below), suggesting similar origins for the response biases observed in both tasks.

**Fig 6:**
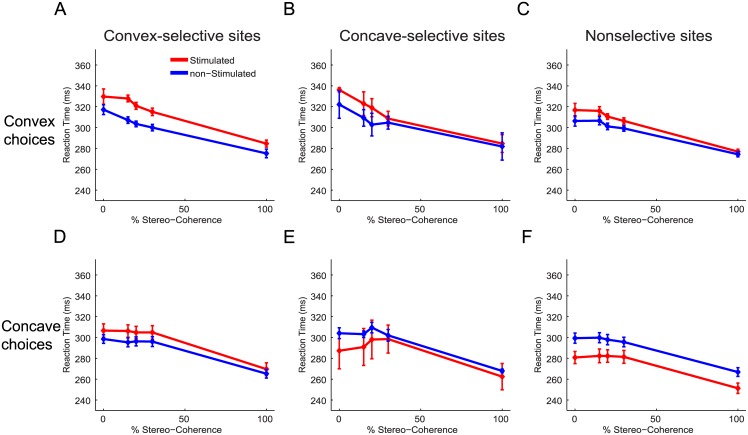
Effect of electrical stimulation in AIP on the reaction times of monkey M2 during 3D-shape categorization. Average reaction times as a function of stereo-coherence for stimulated (red) and non-stimulated (blue) trials. The effect of microstimulation on RTs for convex and concave choices depended on the 3D-shape selectivity of a site. (**A**) Average reaction times for convex choices when stimulating in convex-selective sites. (**B**) Average reaction times for convex choices when stimulating in concave-selective sites. (**C**) Average reaction times for convex choices when stimulating in nonselective sites. (**D**) Average reaction times for concave choices when stimulating in convex-selective sites. (**E**) Average reaction times for concave choices when stimulating in concave-selective sites. (**F**) Average reaction times for concave choices when stimulating in nonselective sites. Error bars indicate ±SEM.

Fig 6 shows the effect of microstimulation on the RTs of monkey M2. Consistent with the data of monkey M1, microstimulation delayed convex and concave choices for convex-selective sites (Fig 6A and 6D; p<0.02 for the main effect of stimulation; p>0.05 for the interaction between stimulation and choice, ANOVA). The size of the delay for convex choices at convex-selective sites was highly dependent on the magnitude of the stimulation-induced bias during the TOAS-task (r = 0.86, p = 0.003, N = 9, Fisher-Z test), suggesting that the interfering processes were pushing the monkey’s actual convex choice in the opposite (concave) direction, thereby causing a delay. No significant correlation was observed between the TOAS-shift and the RT-delay for concave choices at convex-selective sites (r = 0.23, p>0.05), indicating that the delay for concave choices at convex-selective sites may have other origins. Specifically, this suggests that stimulation of convex-preferring neurons induced some 3D-shape related activity that slowed down the upcoming concave choice. In the few concave-selective sites we encountered, we observed a different RT pattern: stimulation-induced delays were on average present for convex choices but small accelerations were observed for concave choices (Fig 6B and 6E). Similarly, for the nonselective sites we noticed that microstimulation delayed convex choices (p = 0.002) while speeding up concave choices (Fig 6C and 6F; p<0.001, ANOVA). Importantly, these qualitatively different RT-patterns displayed by convex- (delays for either choice), concave- and nonselective (faster concave, delayed convex choices) sites demonstrate that the 3D-shape preference of AIP sites in Monkey M2 still somehow influenced microstimulation effects upon RTs during 3D-shape categorization: if the 3D-shape preference of a site were irrelevant and not influencing 3D-shape categorization, all microstimulation effects upon RTs should resemble those of the nonselective sites. Yet, this was not the case.

There was no significant correlation between the choice probability of a site and any of the microstimulation effects on the 3D-shape categorization behavior of monkey M2 (p>0.05).

We have previously shown that microstimulation of 3D-shape selective neurons in the IT cortex of monkey M2 consistently increases the number of preferred choices (Verhoef et al. 2012). One potential explanation for the different microstimulation results between IT and AIP in monkey M2 could be that monkey M2 changed her task strategy from one experiment to the other. Therefore we stimulated in eight 3D-shape selective IT sites after finishing the AIP-stimulation experiment. In agreement with our previous findings, microstimulation in IT significantly increased the number of preferred choices (mean shift = 12%, p = 0.004, t-test; correlation between signed psychometric shift and signed d’ = 0.88, p<0.001), significantly accelerated preferred choices (p = 0.03, ANOVA) and tended to delay nonpreferred choices (mean delay = 4.6ms, p = 0.23, ANOVA). Thus it seems unlikely that a different task strategy underlies the incongruent microstimulation results in AIP of monkey M2. We also varied current strength, ranging between 1μA and 55μA, but these different currents merely attenuated (current<35μA) or amplified (55μA) the concave biases (data not shown). Next, we explored a range of grid positions, spanning 4mm in the anterior-posterior axis, but similarly strong concave biases were observed in all positions. Finally, we trained monkey M2 to indicate the perceived 3D shape by means of an eye movement to choice targets located above or below the stimulus (up-down saccades instead of left-right saccades). Pilot sessions indicated that microstimulation during the TOAS-task with choice targets located along the vertical axis caused minimal response biases. Microstimulation during 3D-shape categorization with a vertical choice-axis did not cause an increase of preferred choices (mean shift = -2.6%, p>0.05, N = 16 selective sites), nor a response bias towards either choice target (mean shift = -1.6%, p>0.05), and no effects on the average RTs (p>0.05). This lack of any average microstimulation effect on 3D-shape categorization can be explained by the smaller and more variable saccade-related biases, as measured during the TOAS-task with vertical choice targets. Microstimulation during the TOAS-task on average increased the proportion of upward choices, the direction of which corresponded to convex choices during 3D-shape categorization (mean shift = 12ms, p<0.001). Importantly, the direction and magnitude of the saccade-related bias during the TOAS-task correlated significantly with the direction and magnitude of the bias during categorization (r = 0.7, p = 0.002, Fisher-Z test, N = 16). Thus stimulation-induced interfering activity was still influencing behavior during the 3D-shape categorization task with choice targets along the vertical axis, but now in a more balanced way. Note that the absence of a stimulation-induced preferred-choice bias in monkey M2 cannot be attributed to any lack of clustering or low 3D-shape selectivity, since the largest clusters and the most sensitive neurons where found in AIP of this monkey (see Fig 2 in [26]).

Electrical microstimulation had generally no effect on the average vergence eye movements of the monkeys in response to the disparity stimuli. Moreover, we did not observe differences in the way microstimulation affected vergence eye movements in each monkey. Hence, differential effects of microstimulation on the vergence eye movements of each monkey do not readily explain the different effects of microstimulation on the categorization behavior of each monkey.

In summary, microstimulation in 3D-shape selective clusters of monkey M2 elicited a strong choice bias unrelated to the 3D-shape preference of the stimulated clusters. Nonetheless, the 3D-shape preference of a site did determine how microstimulation affected the average RTs during 3D-shape categorization as the pattern of stimulation effects on RTs depended on the 3D-shape preference of a site. This indicates that microstimulation in AIP of monkey M2 also activates processes associated with the 3D-shape preference of a site. However, and in contrast to monkey M1, the saccade-related processes dominated the 3D-shape related processes.

## Discussion

We examined the role of macaque cortical area AIP in 3D-shape categorization. Microstimulation in AIP during a 3D-shape-categorization task affected choices (M1) and reaction times (M1 and M2) in a manner that depended on the 3D-shape selectivity of the stimulated site. In addition to 3D-shape-related processes, microstimulation in AIP also induced "saccade-related" processes in both monkeys. The saccade-related processes apparently competed with the 3D-shape related processes, causing preferred choices to be delayed following microstimulation.

Previously we observed large and consistent effects of microstimulation in the IT cortex of the same monkeys that participated in the present study [17]. In comparison to IT, microstimulation in AIP causes relatively small increases in the amount of preferred choices for monkey M1 and no significant preferred-choice bias for monkey M2. Moreover, in contrast to the acceleration of preferred choices induced by stimulation of IT, the execution of preferred choices was generally delayed following AIP microstimulation. Our findings suggest that these differences are at least partly explained by the additional activation of interfering processes in AIP that may have been absent in IT.

AIP is strongly connected to LIP [27] and electrical microstimulation in depth-structure selective patches in AIP activates LIP [34]. Furthermore AIP has some connections to FEF as well [27]. The responses of neurons in both LIP and FEF are modulated by disparity [4,35]. Together, these areas may be part of a parietal-frontal network that analyzes disparity for behavioral purposes, such as perceptual-decision making, object-directed grasping, saccade-target selection, and vergence regulation. Crucially, LIP and FEF are strongly involved in goal-directed saccade behavior, and may have been activated during microstimulation in AIP, thereby causing the observed saccade-related interferences.

Note that while we conveniently designated the interfering stimulation-induced activity ‘saccade-related’, we do not know the exact origin of this biasing activity. In fact, in two sessions of monkey M2 in which significant stimulation-induced response biases were observed during the TOAS task, we unsuccessfully attempted to evoke saccades while the monkey fixated a small dot (200μA, 250–500Hz, 200ms, biphasic pulses of 0.2ms) [36,37]. This suggests that the biasing activity might not be purely saccadic in origin but is merely related to saccades, as in response criterion changes or spatial-attention biases [38,39].

One potential way to avoid the saccade-related interferences is to perform the microstimulation experiment while monkeys report their 3D percepts using another type of motor response, such as pressing two different levers. Nonetheless, in this case it is not certain that the results would not be obscured by similar interferences of a different origin. Specifically, AIP performs a role in object grasping [40] and is located near the parietal-reach area [41]. Moreover electrical microstimulation of AIP activates the nearby reach-related MIP [34]. Stimulating AIP could activate grasp- and reach-related activity, and thereby bias reach-and-grasp responses just as it biased saccadic responses. Thus it is unclear whether choosing a different effector for the response, would lead to microstimulation effects that are more tightly linked to the 3D-shape preference of the stimulated AIP site. Another way to potentially more sensitively measure AIP's contribution to 3D-shape categorization would be to train the monkeys in a task with response contingencies that are not fixed to particular spatial locations (left-right, up-down), e.g. by associating the saccade response for convex and concave shapes with response targets that differ in some feature (e.g. color). This way, saccades indicating convex and concave responses can be evenly distributed across space and dissociated from saccade-direction related effects following microstimulation, thus potentially mitigating some of the adverse effects of stimulation-induced saccade-related activity.

Microstimulation in AIP produced different behavioral outcomes in the two monkeys in our study. Monkey M1 showed clear effects of microstimulation on 3D-shape categorization choices that were predicted by the 3D-shape preferences of the stimulated sites. Monkey M2's results were less clear and were mostly determined by the stimulation-induced saccade-related activity. These different results between monkeys may have resulted from different task strategies, such that monkey M1 relied on the 3D-shape selective activity in AIP, while monkey M2 did not. For example, it is possible that monkey M2 relied more on the 3D-shape selective IT activity [16, 17], and much less on activity in AIP compared to monkey M1. Alternatively, some factors (e.g. stronger co-activation of LIP or FEF) may have hampered our ability to detect monkey M2's reliance on AIP activity. The fact that monkey M2's microstimulation effects on reaction times were dependent on the 3D-shape preferences of the stimulated sites, suggests that monkey M2 did to some degree rely on 3D-shape selective AIP activity. Another cause for the different results between both monkeys may be related to the exact part of AIP that was stimulated. Although we stimulated a range of 3D-shape selective AIP positions in both monkeys, and observed similar effects at each position, we cannot exclude the possibility that some effective 3D-shape selective AIP positions in monkey M2 have been overlooked: stimulating these unexamined AIP sites of monkey M2 might have produced more straightforward results. Indeed, AIP is not a homogeneous area, consisting of regions with different functional specializations: Anterior AIP appears more involved in reaching and grasping, whereas posterior AIP seems more specialized in object processing and is more heavily connected to IT [27, 30, 34, 42]. Small differences in electrode positions between monkeys could have resulted in different amounts of co-activation of IT (see below for further discussion on this topic). Furthermore, in this study we targeted posterior AIP, although we cannot exclude residual electrical spread to anterior AIP. These considerations, and assuming that AIP is primarily involved in object analysis for grasping, raise the possibility that more consistent results between monkeys would be obtained if using a task that involves a grasping component (and stimulating in anterior AIP).

Correlations between the firing rate and reaction times have been observed in several dorsal-stream areas (e.g. MT, MST, VIP) using tasks that involved judgments about stimulus motion [43,44]. We previously observed that stronger firing rates in AIP during 3D-shape categorization were associated with delayed nonpreferred choices and accelerations of choices favoring the preferred shape of a 3D-shape selective site [26]. The relationship between RT and spike rate depended on the magnitude of the choice probability of a site: in sites with the largest choice probabilities higher spike rates were associated with the fastest preferred choices and with the slowest nonpreferred choices. These findings corroborate models in which perceptual decisions are based on weighted evidence originating from opponent neuronal populations, in our case one with convex- and another with concave-preferring neurons, that influence each other’s input into the decision stage via, e.g., feed-forward or lateral inhibition [45]. A previous study applied microstimulation to 3D-shape selective neuronal clusters in IT, and provided additional evidence that such a mechanism underlies 3D-shape categorization [17]. Similarly and in agreement with the opponent-populations model, microstimulation of AIP delayed nonpreferred choices and tended to accelerate preferred choices for sites in monkey M1 with relatively large choice probabilities and smaller saccade-related interferences. These findings provide converging evidence that 3D-shape categorization relies on the activity of opponent pools of neurons.

We observed a strong correlation between the choice probability of a MUA site and the microstimulation effects on the choices made by monkey M1 during 3D-shape categorization. Moreover, stimulation-induced RT delays were also better countered on sites with larger choice probabilities. These findings could have resulted from sites having greater choice probabilities being better monitored by decision-related areas so that activation of the neurons of such an area induced a relatively strong task-related signal. Another non-mutually-exclusive possibility pertains to the density of clustering of 3D-shape selective neurons. Specifically, choice probabilities appear to be larger in clusters of neurons with a similar stimulus preference compared to choice probabilities of neurons outside such clusters [46,47]. In addition, larger microstimulation effects are to be expected if more neurons with a similar shape preference are situated in the electrode’s neighborhood. Consequently the density of clustering may be the underlying factor explaining why microstimulation effects are stronger at sites with higher choice probabilities.

The above findings have interesting implications for our understanding of how information is processed in the parietal cortex. Although we stimulated the same cells during both the discrimination task and the TOAS task, behavioral effects depended on the task at hand. For example, microstimulation of convex-selective sites in area AIP of monkey M1 increased the number of convex choices during 3D-shape discrimination but conversely increased the number of opposite choices, i.e. in the concave direction, during the TOAS task. Additionally, microstimulation of convex-selective sites in monkey M2's AIP delayed concave choices during 3D-shape discrimination but facilitated these same responses, i.e. in the concave direction, during the saccade task (p = 0.028, ANOVA; p>0.05 for M1; data not shown). This observation implies that the information conveyed by 3D-shape selective neurons was used during 3D-shape discrimination but largely or entirely ignored during the TOAS task, even though 3D-shape selective cells were still electrically (but not visually) stimulated during the TOAS task. Since behavior during 3D-shape discrimination depended on saccade-related processes, it seems likely that the activity of saccade-related neurons could not be ignored during this task. In contrast, 3D-shape selective neurons are irrelevant during the saccade task and thus can be safely disregarded. These findings therefore corroborate the view that sensory and motor-related representations coexist partly independently for read-out purposes in parietal cortex [48–51]. Hence, the heterogeneous activity in the intraparietal sulcus is dynamically weighed according to its relevance in a specific context.

Three-dimensional shape perception likely relies on a network of brain areas throughout the brain [1–3]. Thus far we have tested two areas, namely IT [16,17] and AIP [26], for their role in 3D-shape categorization. It should be noted that AIP is both anatomically [27], functionally [42], and effectively [34] connected to those regions in IT that have previously been causally linked to 3D-shape categorization behavior. Hence, we cannot exclude the possibility that some of the microstimulation-induced behavior that was related to the 3D-shape preference of the stimulated AIP sites, was in fact caused by an indirect activation of 3D-shape selective neurons in IT. Alternatively, stimulating AIP could have interfered with 3D-shape related activity in IT, thereby causing the delays in reaction times that we observed.

AIP and IT may be part of different parallel-processing streams with different perceptual specializations. As part of the ventral visual stream, IT could be more involved in perceptual decisions for recognition purposes. Conversely, as part of the dorsal visual stream, activity in AIP might be more relevant for performing object-directed grasping actions. Nonetheless, both areas may still contribute to the performance in both recognition and grasping tasks, although likely not to the same degree. Hence, it is not yet obvious to what extent each area contributes independently of the other to 3D-shape categorization, and how they would cooperate to achieve 3D-shape perception. Future studies are needed to resolve these issues.

Our findings further support the idea that dorsal-stream areas contribute to more traditional perceptual tasks. Evidence from previous studies suggest a role for dorsal-steam areas in such diverse perceptual tasks as motion categorization [21], direction of heading discrimination [19,20], motion-direction discrimination [18], motion detection [43], coarse depth discrimination [22], and discrimination of orientation of disparity-defined surfaces [23]. Here we provide evidence that dorsal-stream area AIP is part of a network involved in 3D-shape categorization.
